# The Kinetics of Enzyme Mixtures

**Published:** 2014-03

**Authors:** Simon Brown, Noorzaid Muhamad, Kevin C Pedley, David C Simcock

**Affiliations:** 1 Deviot Institute, Deviot, Tasmania 7275, Australia,; 2 School of Human Life Sciences, University of Tasmania, Launceston, Tasmania 7250, Australia; 3 Royal College of Medicine Perak, University Kuala Lumpur, Ipoh, Malaysia; 4 Institute of Food, Nutrition and Human Health, Massey University, Palmerston North, New Zealand; 5 Faculty of Medicine, Health and Molecular Sciences, James Cook University, Cairns, Queensland 4870, Australia

**Keywords:** aspartate aminotransferase, enzyme kinetics, heterogeneity, isozyme

## Abstract

Even purified enzyme preparations are often heterogeneous. For example, preparations of aspartate aminotransferase or cytochrome oxidase can consist of several different forms of the enzyme. For this reason we consider how different the kinetics of the reactions catalysed by a mixture of forms of an enzyme must be to provide some indication of the characteristics of the species present. Based on the standard Michaelis-Menten model, we show that if the Michaelis constants (*K*_m_) of two isoforms differ by a factor of at least 20 the steady-state kinetics can be used to characterise the mixture. However, even if heterogeneity is reflected in the kinetic data, the proportions of the different forms of the enzyme cannot be estimated from the kinetic data alone. Consequently, the heterogeneity of enzyme preparations is rarely reflected in measurements of their steady-state kinetics unless the species present have significantly different kinetic properties. This has two implications: (1) it is difficult, but not impossible, to detect molecular heterogeneity using kinetic data and (2) even when it is possible, a considerable quantity of high quality data is required.

## INTRODUCTION

Proteins are intricate structures, often requiring complex post-translational modification to generate the mature form, that are eventually recycled [1]. Consequently, even a population of mature molecules can be expected to include both newly synthesised and older molecules that may exhibit some variation in activity [2, 3]. For example, an iron-sulphur centre is important in binding citrate by aconitase (E. C. 4.2.1.3) and therefore in the catalytic function of the enzyme [4], but the apoprotein is involved in iron-sensing and influences the supply of iron for the synthesis of the prosthetic group of the mature enzyme [4, 5]. There are many other examples of the loss of prosthetic groups from enzymes, such as the loss of of pyridoxal 5′-phosphate from aspartate aminotransferase (E. C. 2.6.1.1) [6], of Mg^2+^ from the active site of ribulose 1,5-bisphosphate carboxylase/oxygenase (rubisco, E. C. 4.1.1.39) [7] or of Cu_B_ from cytochrome *bo* (E. C. 1.10.3.10) in *Escherichia coli* grown in a low-copper medium [8]. In some cases, the loss of a group not necessarily directly involved in the reaction can modify the kinetics of an enzyme. For example, bovine cytochrome oxidase usually contains 3-4 molecules of cardiolipin per enzyme molecule and their removal can alter the kinetics of the enzyme [9].

In addition to the molecular heterogeneity associated with the normal lifecycle of proteins, variation associated with oligomerisation can be significant. First, some homo-oligomeric enzymes can comprise different combinations of subunits, which may have different kinetic properties. For example, various combinations of subunits can be observed for the tetrameric lactate dehydrogenase (E. C. 1.1.1.27) [10, 11]. This is distinct from the tissue-specific isoforms of some enzymes in which there is a difference between tissues, but a single isoform tends to be present in each. Second, some enzymes may undergo changes in the extent of oligomerisation in response to changes in conditions. For example, glyceraldehyde 3-phosphate dehydrogenase (E. C. 1.2.1.12) is monomeric or homotetrameric depending on the conditions and has different kinetics in the two states [12].

Other factors contributing to the apparent heterogeneity of enzymes include the presence of more than one isozyme catalysing a single reaction. For example, different isozymes catalyse the malate dehydrogenase (E. C. 1.1.1.37) and aspartate aminotrans- ferase reactions in the mitochondria and cytosol [2], distinct from the case of aconitase in which the same holoenzyme is found in both spaces. In a small number of cases, a single enzyme catalyses two different reactions using the same substrate. For example, rubisco can carboxylate or oxygenate the substrate and so the two reactions are competitive in the presence of both CO_2_ and O_2_ [13]. Finally, there are instances in which enzyme molecules can be associated with other enzymes in multienzyme complexes or with other intracellular structures. For example, glutamate dehydrogenase (E. C. 1.4.1.3) has been reported to be associated with the endoplasmic reticulum [14], with the lysosomal membrane [15] and even with mRNA [16], and it may be part of a multienzyme complex [17], and lipase (E. C. 3.1.1.3) is activated on binding to a lipid interface [18].

The intrinsic heterogeneity of proteins is rarely considered in analyses of enzyme kinetics, but it prompts several questions, of which we address two. First, how different do two isoforms have to be to facilitate the identification and characterisation of the heterogeneity of a population of enzymes? Second, how might heterogeneity be detected in the kinetics of an enzyme?

## BACKGROUND MATERIALS

The most common description of the kinetics of an enzyme (E) converting a substrate (S) to a product (P) by way of an enzyme-substrate complex (C) is the Michaelis-Menten mechanism

$S+E{↔}_{k_{-1}}^{K_{1}}C{↔}_{k_{-2}}^{K_{2}}P+E$

(1)

[19], where the *k*_i_s are rate constants. The rate equation for this reaction when the concentration of P is negligible is

(2) v=Vmax SKm+S,

where *s* is the concentration of S, *V*_max_ is the maximum rate of reaction and *K*_m_ is the Michaelis constant [20]. The usual statements of this model do not contemplate that the second step in (1) is reversible, indeed, it is often suggested that it cannot be reversed which is inappropriate for many biochemical reactions. Furthermore, it is not possible for an enzyme catalysing (1) to behave as a Michaelis-Menten enzyme in both directions [21]. For reasons that will become apparent, we have departed from this practice, but the standard analysis of (1) does apply when the concentration of P is negligible, which would preclude any appreciable reverse reaction. There is some question as to the appropriateness of (1) [22] and more complicated behaviours have been reported [23].

**Constraints on equilibrium constants:** Irrespective of the state of the enzyme the equilibrium constant *K* of (1) is

(3) $$K=K_{1}K_{2}=\frac{k_{1}k_{1}}{k_{-1}k_{-2}}$$

where *K*_1_ and *K*_2_ are the equilibrium constants of the first and second steps, respectively, of (1). However, the enzyme-substrate complex (C) may be modifed by the changing state of an enzyme, because any change affecting the substrate binding site may alter the rate constants and, therefore, the binding affinity and the catalysis. In such cases *K*_1_ and *K*_2_ may be changed, but *K* will not. Similarly, the enzyme-substrate complex (C) is likely to differ between two enzymes even if they catalyse the same reaction. So, *K*_1_ and *K*_2_ may vary among the various forms of an enzyme, but any change in one must be associated with an inverse change in the other. 

Both *K*_m_ and *V*_max_ [20] can be expressed in terms of these equilibrium constants

(4) $$K_{m}=\frac{k_{-1}+k_{2}}{k_{1}}=\frac{1}{K_{1}}+\frac{k_{-2}}{k_{-1}}\frac{k_{2}}{K_{1}}=\frac{K_{2}}{K}(1+\emptyset K_{2})$$

And 

(5) $$V_{\max}=k_{2}E_{t}=k_{-1}\emptyset K_{2}E_{t}$$

Where $k_{-1}^{'}\emptyset^{'}=k_{-1}^{'}$ and *E*_t_ is the total enzyme concentration. Of course, *V*_max_∝*K*_2_ whereas *K*_m_∝*K*_2_^2^, which implies that the latter might be a more sensitive indicator of the presence of modified enzymes than the former. While both *K*_m_ and *V*_max_ increase with *K*_2_ (4-5), the catalytic efficiency of an enzyme [24, 25]

(6) $$n=\frac{V_{\max}}{K_{m}E_{t}}=\frac{k_{-1}\mathrm{\emptyset K}}{1+\emptyset K_{2}}$$

is decreased as *K*_2_ rises, consistent with the depletion of the enzyme-substrate complex [26].

The *V*_max_ of a modified enzyme ($V_{\max}^{'}$) compared with that of the standard enzyme (*V*_max_) is 

$\alpha =\frac{V_{\max}^{,}}{V_{\max}}=\frac{k_{-1\emptyset^{,}K_{2}^{,}}^{,}}{k_{-1}\emptyset K_{2}}\approx \frac{K_{2}^{,}}{K_{2}}$

(7)

where it is assumed that $k_{-1}^{'}\emptyset^{'}=k_{-1}^{'}$ or $k_{-2}^{'}=k_{-2}$. Similarly, the corresponding comparison of the *K*_m_s of the same two forms is 

(8) $$\beta =\frac{K_{m}^{,}}{K_{m}}=\frac{K_{2}^{,}(1+\emptyset^{,}K_{2}^{,})}{K_{2}(1+\emptyset K_{2})}$$

and, if $k_{-1}^{,}\approx k_{-1}$,

$\beta \approx \frac{\emptyset K_{2}}{1+\emptyset K_{2}}\alpha^{2}+\frac{1}{1+\emptyset K_{2}}\alpha ,$

(9)

which also implies that *β* ≤ *α*^2^ if *α* ≥ 1, where equality holds if *α* = 1. So the relative catalytic efficiency of the two forms of the enzyme is just

(10) $${ŋ}_{\mathrm{rel}}=\frac{{ŋ}^{´}}{ŋ}=\frac{V_{\max}^{´}K_{m}}{V_{\max}K_{m}^{´}}=\frac{\alpha }{\beta }$$

and using (9) $n_{\mathrm{rel}}\approx 1/(\mathrm{qa}+1-q)$, where $q=\emptyset k_{2}/(1+\emptyset k_{2})$<1.

Order of magnitude estimates for some of these parameters can be obtained from the work of Bar-Even *et al.* [27] who analysed kinetic data obtained from BRENDA [28]. Based on their analysis, each of *k*_2_, *K*_m_ and n has a roughly lognormal distribution with median values of 13.7 s^-1^, 0.13 mM and 1.25 × 10^5^ M^-1^ s^-1^, respectively. Approximately 60% of all reactions had *k*_2_ ≈ 1-100 s^-1^, *K*_m_ ≈ 0.01-1 mM and n ≈ 10^3^-10^6^ M^-1^ s^-1^, but the real ranges were considerably larger. While these values represent many enzymes from many species, they do provide some indication of what might be possible.

**Figure 1:**
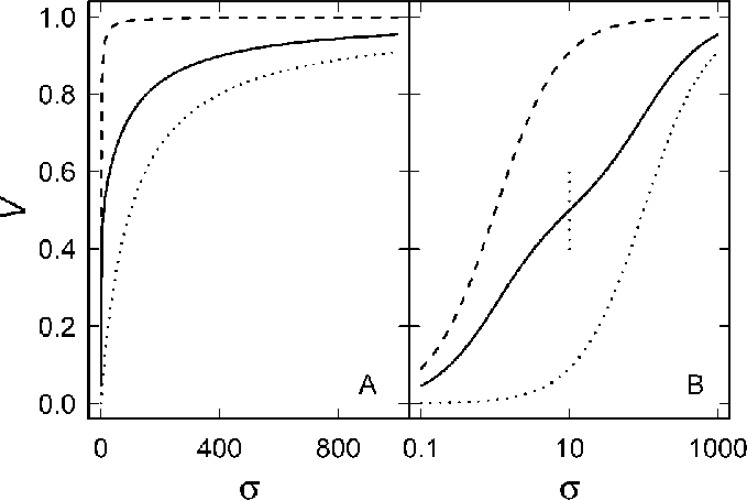
Kinetics of a mixture of two enzymes (13) and of the two component enzymes (1). It was assumed that *α* = 1 and *β* = 100, and curves are plotted for *p* = 1 (– – – – –), *p* = 0.5 (———) and *p* = 0 (∙∙∙∙∙∙∙∙). The vertical dotted line in (B) indicates the value of *σ*_1_ at which the second term of *dV*/*dσ* vanishes (15). The same values are plotted in (A) and (B), but note the logarithmic scale of the abscissa in (B)

**The simplest case:** We consider a mixture of two forms of an enzyme to one of which (2) applies and the other has modified *V*_max_ and *K*_m_. This can be written

(11) $$V=p\frac{V_{\max^{S}}}{K_{m}+S}+(1-p)\frac{V_{\max}^{´}S}{K_{m}^{´}+S}$$

where *p* is the fraction of molecules in the form with kinetics described by (2). Using *α* ≥ 1 (7) and *β* (8) and letting $=s/K_{m}$ , the non-dimensional form is

(12) $$V=\frac{v}{V_{\max}}=\left(p\frac{1}{1+\sigma }+(1-p)\frac{\alpha }{\beta +\sigma }\right)\sigma$$

(Figure 1A) which can be written as the sum of a term that is independent of *p* and another that is not

$V=\frac{\alpha \sigma }{\beta +\sigma }+p\left(\frac{1}{1+\sigma }-\frac{\alpha }{\beta +\sigma }\right)\sigma$

(13)

Of course, the apparent *V*_max_ and *K*_m_ for the mixture are $(p+\left(1-p\right)a)V_{\max}$ and, if *p* = 0.5,$\sqrt[{2}]{\beta }$ , respectively. The second term can be positive or negative and it vanishes when σ=(β-a)/(a-1) if *α* ≠ 1, and it is always positive if (1 + *σ*) < (*β* + *σ*)/*α*. This term can cause significant deviation from standard Michaelis-Menten behaviour (Figure 1B). So, at low *σ* *V* is greater than the first term on the RHS and it may be smaller at higher *σ* depending on the magnitudes of *α* and *β* (Figure 1B). It is clear from the derivatives of *V*

(14) $$\frac{d^{n}V}{d\sigma^{n}}=({-1)}^{n+1}n!\left(\frac{\alpha \beta }{{\left(\beta +\sigma \right)}^{n+1}}+\left(\frac{1}{\left(1+\sigma \right)}-\frac{\alpha \beta }{{\left(\beta +\sigma \right)}^{n+1}}\right)P\right)$$

that the second term vanishes when

$\sigma_{n}=\frac{\beta -({\alpha \beta )}^{1/(n+1)}}{-1+({\alpha \beta )}^{1/(n+1)}}$

(15) .

It is apparent from Figure 1B that there is a minimum in *dV*/ln *σ*

$\frac{\mathrm{dV}}{\mathrm{dIn\sigma}}=\sigma \frac{\mathrm{dV}}{\mathrm{d\sigma}}=\left(\frac{\alpha \beta }{({\beta +\sigma )}^{2}}+\left(\frac{1}{({1+\sigma )}^{2}}-\frac{\alpha \beta }{({\beta +\sigma )}^{2}}\right)P\right)\sigma$

(16)

at $\left(\beta \sqrt[{2}]{P}-\sqrt[{2}]{\alpha \beta })/(-1+\sqrt[{2}]{\alpha \beta \left(1-p\right))}\right)$ that separates maxima approaching 

And

${\left.\frac{\mathrm{dV}}{\mathrm{d In \sigma}}\right|}_{\sigma =1}=\frac{\alpha \beta }{{\left(\beta +1\right)}^{2}}+\left(\frac{1}{4}-\frac{\alpha \beta }{{\left(\beta +1\right)}^{2}}\right)P$

(17)

(18) $${\left.\frac{\mathrm{dV}}{\mathrm{d In \sigma}}\right|}_{\sigma =\beta }=(\frac{\alpha }{4\beta }+\left(\frac{1}{{\left(\beta +1\right)}^{2}}-\frac{\alpha }{4\beta }\right)P)\beta$$

as *β* increases. The depth of minimum in *dV*/ln *σ* between these peaks (δ) can be estimated from the difference in the coordinates of the line joining the peaks 

$f\left(\sigma \right)=\left({\left.\frac{\mathrm{dV}}{\mathrm{d In \sigma}}\right|}_{\sigma =\beta }-{\left.\frac{\mathrm{dV}}{\mathrm{d In \sigma}}\right|}_{\sigma =1}\right)\frac{\mathrm{In\sigma}}{\mathrm{In\beta}}+{\left.\frac{\mathrm{dV}}{\mathrm{d In \sigma}}\right|}_{\sigma =1}=\left(\frac{{\left(\beta -1\right)}^{2}}{4({\beta +1)}^{2}}\left(\left(1-p\right)\alpha -p\right)\right)\frac{\mathrm{In\sigma}}{\mathrm{In\beta}}+\frac{\alpha \beta (1-p)}{({\beta +1)}^{2}}+\frac{p}{4}$

(19)

and *dV*/ln *σ* at the minimum

**Figure 2:**
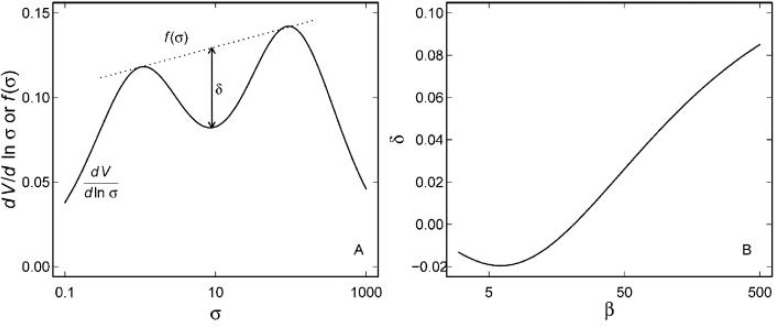
The derivative of (13) and the definition of the depth (*δ*) of the trough (A) and the variation of *δ* with *β* (B). In (A), *α* = 1, *β* = 100 and *p* = 0.4, and the derivative (———) and *f*(*σ*) (∙∙∙∙∙∙∙∙) are given by (16) and (19), respectively. In (B), *α* = 1 and *p* = 0.5, and *δ* is given by (20).

$\delta =f\left(\frac{\beta \sqrt[{2}]{p}-\sqrt[{2}]{\alpha \beta }}{-1+\sqrt[{2}]{\alpha \beta \left(1-p\right)}}\right)-\frac{\mathrm{dv}}{\mathrm{d In \sigma}}\left|1_{\sigma =\frac{\beta \sqrt[{2}]{p}-\sqrt[{2}]{\alpha \beta }}{-1+\sqrt[{2}]{\alpha \beta \left(1-p\right)}}}\right.$

(20)

(Figure 2A). If *β* is less than about 20 there is no trough, but *δ* increases for larger values of *β* (Figure 2B). Integration of (13) yields a complex expression for the progress curve. There is no obvious indication from this that two different forms of the enzyme are present, irrespective of the magnitudes of *α*, *β* or *p*. It appears that the number of forms of the enzyme might be more apparent from higher derivatives (14), but this approach would require a considerable quantity of high quality data.

**Figure 3:**
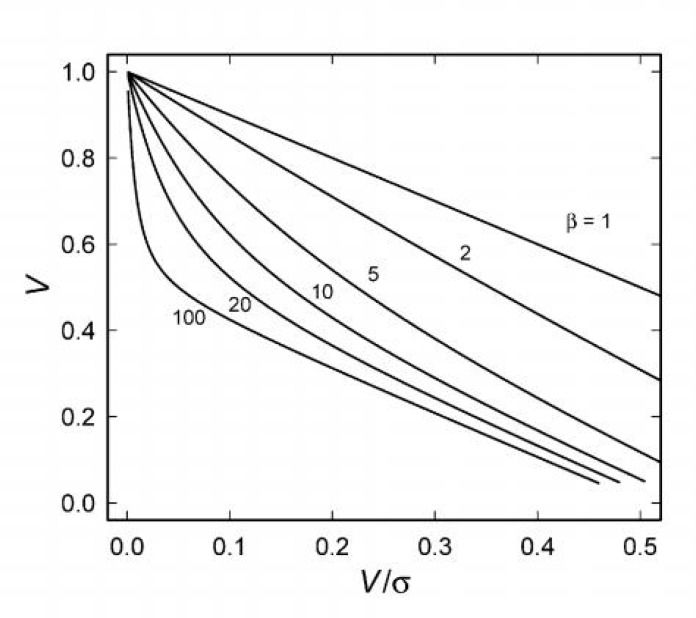
An Eadie-Hofstee plot of (13) for several values of *β*. In each case *α* = 1 and *p* = 0.5.

A common approach to the analysis of kinetic data is to linearise (2) by transforming the data. While transformation introduces bias into parameter estimates, it might be helpful here. Arguably, the most reliable of the transformation is the Eadie-Hofstee transform [29]. Applying this to (12) yields curves that are distinctly nonlinear (Figure 3). Even in this case, however, *β* must exceed about 20 for the kinetics of the two forms of the enzyme to be apparent. However, the curves shown in Figure 3 could also arise from a single isozyme with non-Michaelis-Menten kinetics [23].

**Figure 3:** An Eadie-Hofstee plot of (13) for several values of *β*. In each case *α* = 1 and *p* = 0.5.

**The general case:** If there are several forms of an enzyme, whether because of differences in age, oligomerisation, history or exposure to effectors, then (13) can be extended to

$V=\sum_{i=1}^{n}P_{i}\frac{\alpha_{i}\sigma }{\beta_{i}+\sigma }$

(21)

where ∑_i_*p*_i_ = 1 is a distribution function and *α*_i_ and *β*_i_ are dimensionless multiples of the smallest *V*_max_ and its associated *K*_m_, respectively, so *α*_1_ = *β*_1_ = 1 and *α*_i_ ≥ 1 for *i* > 1. As for the simple model, *p*_i_ and *α*_i_ are confounded unless the *p*_i_ can be varied.

The derivatives of (21) are just

$\frac{\mathrm{dv}}{\mathrm{d In\sigma}}=\sum_{i=1}^{n}P_{i}\frac{\alpha_{i}\beta_{i}\sigma }{(\beta_{i}+{\sigma )}^{2}}$

(22)

and, 

(23) $$\frac{d^{2}v}{{d\left(\mathrm{In\sigma}\right)}^{2}}=\sum_{i=1}^{n}p_{i}\frac{\alpha_{i}\beta_{i}\left(\beta_{i}-\sigma \right)\sigma }{(\beta_{i}+{\sigma )}^{3}}$$

so there is an oscillation associated with each *β*_i_, analogous to those shown for the simplest case in Figures 1B and 2. For a mixture of *n* isozymes the limiting value of *V* is just the weighted average$\sum_{i=1}^{n}p_{i}\alpha_{i}$. There is no similar simple expression for the apparent *K*_m_ but we conjecture that it is related to the weighted geometric mean (∏i=1npiαiβi/∏i=1npiαi)1n .

**Application to aspartate aminotransferase:** Aspartate aminotransferase catalyses the reversible transfer of an amino group from aspartate to α-ketoglutarate to yield glutamate and oxaloacetate. Isozymes are present in both the mitochondrial matrix and in the cytoplasm and it has been reported that there are several isoforms of the enzyme in each of the subcellular spaces [2, 30-32]. Consequently, even a purified preparation of one of the isozymes can be heterogeneous.

Even considering just one of each of the cytoplasmic and mitochondrial forms of the enzyme (and ignoring the isoforms of each of them), it is unlikely that a mixture of them could be detected using kinetic data. The kinetic properties of mitochondrial and cytoplasmic isozymes from rabbit liver [33] are shown in Table 1. Clearly, *α* ≈ 1, as we have assumed (although this need not always be the case), and, while *β* = 23 for *α*-ketoglutarate, it is 0.49 for aspartate. It might just be possible to detect contamination of the two isozymes from an Eadie-Hofstee transformation of the activity dependence on the concentration of *α*-ketoglutarate (Figure 3), but this would require a significant quantity of high quality data. It would not be possible by varying the concentration of aspartate (Figures 2B and 3). Since the kinetics of the isoforms that we have not considered are even more similar to each other than are those of the cytoplasmic and mitochondrial isozymes [32], there is little likelihood of observing any indication of this heterogeneity in the kinetic data.

## RESULTS AND DISCUSSION

While considerable emphasis is placed on working with purified enzymes, even the most carefully prepared enzymes are likely to be hetereogeneous. For example, cytochrome oxidase (E. C. 1.9.3.1) preparations often contain at least two forms [3] and, as we have pointed out, there are several isoforms of aspartate aminotransferase in both the mitochondria and the cytoplasm [2, 30-32]. We have shown that the heterogeneity is not apparent from the kinetics of the enzyme unless *β*_i_ > 20. In practice, separating *p*_i_ from *α*_i_ is impossible unless the proportions can be varied or one of the *α*_i_s is known. Consequently, molecular heterogeneity may often be ignored with the result that (2) is used when (21) might be more appropriate. In effect, this represents a problem of estimating the number of isoforms, their proportions and the set of parameters characteristic of each. The statistical problem [34] is not trivial and it requires a considerable quantity of high quality data. 

We have considered the simplest case for two isozymes (11) and a more general case (21), a form of which was contemplated, but not analysed, by Haldane [35]. The simplest case (11) has previously been applied to cytochrome oxidase [36], although no consideration was given to the value of *p*, despite strong evidence that there are at least two forms of the enzyme [3]. More complicated expressions have also been developed to describe the kinetics of cytochrome oxidase [37] to account, in part, for the molecular heterogeneity.

While it is likely that most, if not all, preparations of an enzyme are heterogeneous, whether as a result of the presence of a mixture of lifecycle stages, of isozymes or other forms, it is rarely explicitly considered. Consequently, the impact of the mixture on the kinetics tends to be ignored. We have shown that this is probably reasonable unless *β* > 20, but it does highlight the point that the purity of an enzyme preparation is relative. It might be possible to work with a truly homogeneous preparation in some cases, but in other circumstances a mixture may be the only material available. This prompts one to ask just how many different forms are acceptable and whether there are some forms that might render a mixture unacceptable when it is not possible to identify the heterogeneity from the kinetics alone. 
